# Comparative Transcriptome Analysis Reveals *OsBGs* and *OsGSLs* Influence Sugar Transport through Callose Metabolism under Heat Stress in Rice

**DOI:** 10.3390/ijms24043175

**Published:** 2023-02-06

**Authors:** Ruiwei Luan, Jingyang Liu, Longxing Tao, Guanfu Fu, Caixia Zhang

**Affiliations:** 1 Key Laboratory of Crop Biology of China, Shandong Agricultural University, Taian 271018, China; 2 National Key Laboratory of Rice Biology, China National Rice Research Institute, Hangzhou 310006, China

**Keywords:** rice, heat stress, *BGs* and *GSLs*, callose metabolism

## Abstract

Heat or high temperature stress have caused huge damage to many crops and have become the largest threat in terms of the future. Although a huge amount of research has been conducted to explore the mechanisms of heat tolerance and many achievements were accomplished, the mechanism by which how heat stress (HS) influences the yield is still unclear. In this study, RNA-seq analysis indicated that nine 1,3-β-glucanases (*BGs*) belonging to the carbohydrate metabolic pathway were expressed differently during heat treatment. Therefore, we identified the BGs and glucan-synthase-likes (*GSLs*) in three rice ecotypes and processed the analyses of gene gain and loss, phylogenetic relationship, duplication, and syntenic relationship. We found the possibility of an environmental adaption based on *BGs* and *GSLs* during evolution. Submicrostructure and dry matter distribution analysis confirmed that HS might block the endoplasmic sugar transport pathway by increasing callose synthesis, which may lead to decreased yield and quality in rice production. This study provides a new clue regarding rice yield and quality under HS and provides guidance to rice cultivation and heat tolerance breeding.

## 1. Introduction

As the climate continues to warm, extreme heat events are expected to worsen in severity, frequency, and duration (IPCC, 2014). Extreme global warming could pose serious damage to most crops, becoming the biggest threat to agriculture [1,2,3]. Moreover, the area of cultivated land has decreased sharply, and food security has become increasingly serious [4]. As an important food crop, rice feeds more than half of the world’s population and provides energy for 21% of the world’s population and more than 76% of the population in Southeast Asia [5]. In view of the rising food demand and security risks, increasing production through effective production methods is urgently needed [6].

Heat stress (HS) could cause multiple negative effects on plant morphology, physiology, biochemistry, and molecular pathways at all vegetative and reproductive stages [7,8,9,10]. Temperatures above the optimum temperatures for cultivation are defined as constituting HS. The negative effects of HS include the release of pollens at anthesis stages. Previous studies have demonstrated that spatiotemporally regulated callose deposition and degradation are critical for functional pollen production [11,12,13,14]. The *Arabidopsis* genome encodes 12 callose synthesis genes that are termed glucan-synthase-like (*GSL*) [15] or callose synthase (*CalS*) [16]. Generally, callose levels are regulated by two antagonistic actions: callose synthesis and degradation [17].

Rice yield and quality have been adversely affected by extreme high-temperature weather in recent years. High temperature decreases the kernel weight, root number, total length, and metabolic activities of rice. In future climates, rice crops will be frequently exposed to water deficit and HS at the most sensitive flowering stage, causing spikelet sterility and yield losses. Moreover, photoassimilates are detained in vegetation organs under high-temperature conditions, leading to an increase in the dry matter weight of stem sheath and leaf and a decrease in panicle [10].

Crop yield is closely related to the accumulation, distribution, and transportation of photoassimilates from leaf to grain [12,18,19,20,21]. A stream of sucrose is loaded into molecular sieve tube members; transported through a series of vascular system of the leaves, stems, and caryopsis; and finally unloaded in the grain [22,23,24,25]. Therefore, a decrease in grain weight under high-temperature stress is closely related to the decrease in sucrose transport capacity from source to grain sink [26,27].

In this study, we surveyed the expression level of a low-tolerance accession of rice (i.e., high-temperature susceptibility (HTS) mutant), identified the key genes related to the HS, and explored the mechanism of yield reduction during high temperature. This study provides a new insight into the influence of callose biosynthesis during yield formation under HS and provides new guidance to heat-tolerant breeding and cultivation in the rice industry.

## 2. Results

### 2.1. Transcriptome Analysis of Rice under Heat Stress

A material named HTS (weak high-temperature tolerance) was used in this study to explore the mechanism of yield formation under high temperature in rice. Seedlings with 4–5 leaves were divided into two groups: high-temperature treatment (40 °C from 09:00 to 16:00, and 30 °C from 16:01 to 8:59 of the next day for 6 days) and control (CK; 30 °C from 09:00 to 16:00, and 24 °C from 16.01 to 8:59 of the next day for 6 days). The transcriptomes of the CK and high-temperature groups were sequenced by the Illumina Hiseq 4000 platform. Finally, we obtained ≈338 M clean reads with an average of 8.45 Gb for each sample (Table S1). The data were quality controlled again by Trimmomatic and then were mapped to the reference genome of *Oryza sativa* subsp. *japonica* (V7). The mapped rate was 79.03% on average.

**Figure 1:**
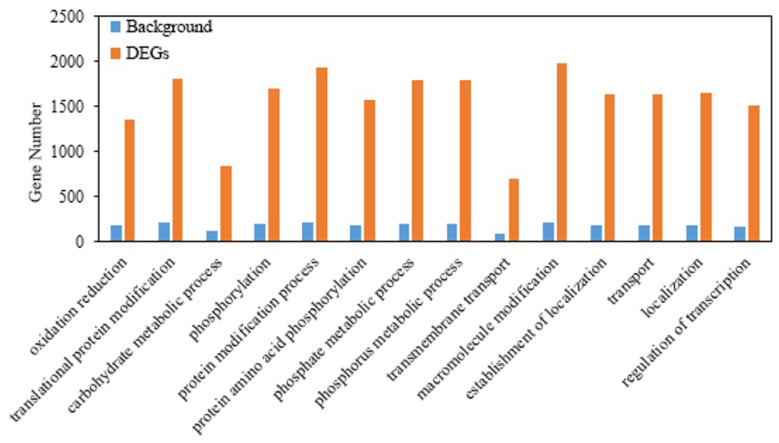
GO enrichment analysis of the DEGs of HTS leaves under heat stress. The blue bars are the gene numbers of the background, and the orange bars are the genes numbers of the DEGs.

On the basis of the RNA-seq analysis, we identified 3032 differentially expressed genes (DEGs), including 1615 upregulated and 1417 downregulated DEGs, during high-temperature treatment. In order to obtain a global landscape of these DEGs, Gene Ontology (GO) enrichment analysis was performed to obtain a global landscape of the DEGs (Figure 1). Among these DEGs, we found that 116 genes were enriched in the carbohydrate metabolic process item and that 9 DEGs belong to the 1,3-β-glucanase (*BG*) family. Previous studies indicated that *BGs* could lead to callose deposition in the plasmodesmata and inhibit carbohydrate transport [9]. For this reason, we believed that callose might play an important role in yield formation under high temperature. Therefore, we processed a comparative analysis of the *BG* and glucan synthase/callose synthase (*GSL*) genes in the genomes of three rice ecotypes.

### 2.2. Identification of the BG and GSL Families in Three Oryza Ecotypes

On the basis of the hidden Markov model (HMM) and Blast scan, 212 *BGs* and 33 *GSLs* in three *Oryza* ecotypes were identified. Among them, 81 *BGs* belonged to *O. sativa* subsp. *indica* (*OS*), 70 *BGs* belonged to *O. sativa japonica* (*Os*), 61 *BGs* belonged to *Oryza glaberrima* (*Og*), 13 *GSLs* belonged to *Os*, 12 *GSLs* belonged to *OS*, and 8 *GSLs* belonged to *Og* (Table S2). Among the three ecotypes, *OS* contained the most *BGs*, *Os* contained the most *GSLs*, and *Og* contained the least *GSLs* and *BGs*.

### 2.3. Phylogenetic Analysis of BG and GSL Families

According to the phylogenetic tree, *OsBGs* can be classified into six clades. The largest clade was Clade III, and the Clade III of *Os* lacked five members compared with those of *Og* and *OS*, which had 23 members. The smallest clade was Clade VI, wherein *Os*, *Og*, and *OS* had two, three, and five members, respectively. *OsGSLs* can be classified into three clades. The biggest clade was Clade II, and five members were found in every *Oryza* ecotype. In Clade I, *Os*, *Og*, and *OS* had three, three, and five members, respectively. No members were found in Clade III of *Os*.

**Figure 2:**
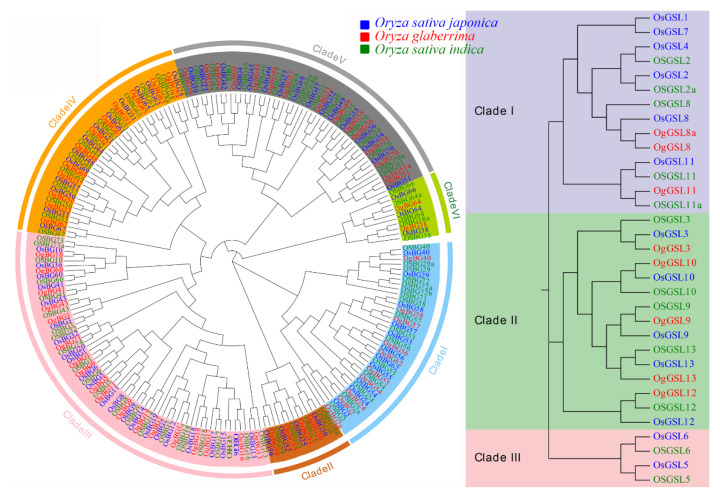
Phylogenetic analysis of *BG* and *GSL* families. The blue genes belong to *Oryza glaberrima*, the red genes belong to *Oryza sativa* subsp. japonica, and the green genes belong to *O. sativa* subsp. *indica*. The different colors of the tree area represent the different clades of the tree.

According to the phylogenetic analysis, we also found that *BGs* were highly conserved in rice. Most genes had at least one orthologous gene in each ecotype. Additionally, we also found 20 *BG* gene expansion or loss events in the three ecotypes (Figure 2) including 10 *BG* expansion events and 4 BG loss events in *Os*. In the *GSL* family, Clade II was also highly conserved. Each member of this clade could find a proper orthologous gene, and no expansion or loss events occurred. In the whole family, each ecotype contained one expansion event, and four gene loss events occurred in *Os*. Briefly, the members of the two families were highly conserved, but their gene expansion and loss also occurred during environmental adaption and evolution (Figure 2). 

### 2.4. Segments and Tandem Duplication Events Involved in the Expansion of the BG and GSL Families

**Figure 3:**
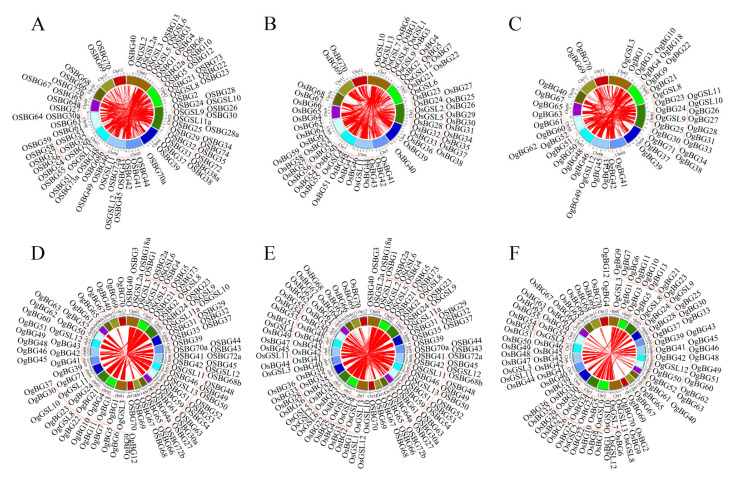
Segment duplication events and syntenic analyses of *BGs* and *GSLs* within and between ecotypes. (**A**–**C**) Segment duplication events related to *BGs* and *GSLs* occurred in the three ecotypes’ genomes: (**A**) *O. sativa* subsp. *indica*, (**B**) *O. sativa* subsp. *japonica*, and (**C**) *O. glaberrima*. The red lines imply the segment duplication events related to the *BG* or *GLS* members. (**D**–**F**) Syntenic blocks between two ecotypes. The red lines represent the syntenic blocks containing *BGs* or *GSLs*.

Several studies indicated that segment duplication and tandem duplication could contribute to the gene expansion [28]. Therefore, we surveyed the family genes involved in segment and tandem duplication events. The results indicated that *OSGSLs* and *OSBGs* were involved in 612 segment duplication events, *OsGSLs* and *OsBGs* were involved in 678 segment duplication events, and *OgGSLs* and *OgBGs* were involved in 371 segment duplication events (Figure 3A–C). Tandem duplication also contributed to the expansion of *BG* families (Table S1). In total, 40 *BGs* (18.87%) were involved in tandem duplication events, but no *GSLs* were involved in tandem repeat events.

Segment duplications contributed greatly to the expansion of the *GSL* and *BG* families. A total of 11 (91.67%, *OS*), 10 (76.92%, *Os*), and 6 (75%, *Og*) *GSL* genes were involved in the segment duplication events, and 70 (86.42%, *OS*), 65 (92.86%, *Os*), and 42 (68.85%, *Og*) *BG* genes were involved in segment duplication events (Table S1). Synteny analysis among the three ecotypes indicated that the synteny blocks were still highly conserved; each block that contained *BG* and *GSL* families can find their proper orthologous blocks in the other two ecotypes (Figure 3D–F).

### 2.5. Expression Pattern of OsBGs and OsGSLs under Heat Stress

**Figure 4:**
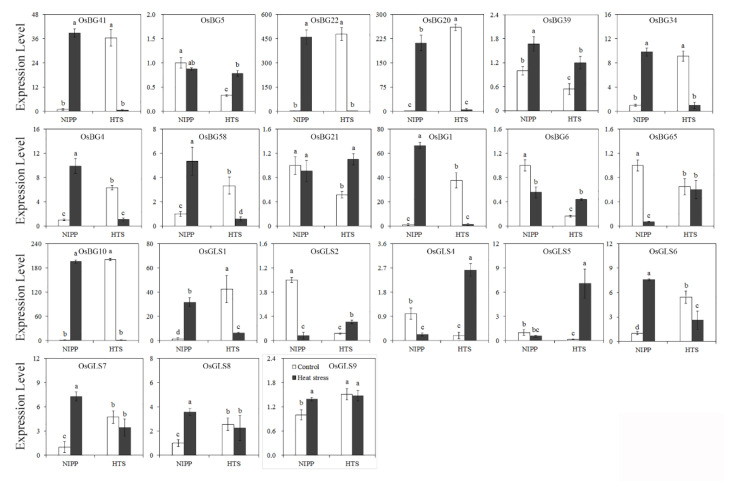
Expression of *BGs* and *GSLs* in two different ecotypes under heat stress. Vertical bars denote standard deviations (n = 3). Different letters indicate significant differences between the control and heat stress within one genotype (*p* ≤ 0.05).

The expression levels of *BGs* and *GSLs* in two rice varieties with different HS tolerance levels were identified to determine the responses of *OsBGs* and *OsGSLs* to HS compared with natural conditions. Most of the relative expression rates of *OsBG* genes measured under HS were different from their respective controls (Figure 4). High levels of *OsBG* expression were induced in Nipponbare (NIPP) leaves by HS. By contrast, in the leaves of HTS, *OsBG* genes, particularly *OsBG1*, *OsBG4*, *OsBG10a*, *OsBG20*, *OsBG22*, *OsBG34*, *OsBG41*, and *OsBG58*, were downregulated considerably. A part of the expression pattern of the *OsGSLs* under HS was similar to those observed in *OsBGs*. In response to the HS, the *OsGSL1*, *OsGSL6*, *OsGSL7*, *OsGSL8*, and *OsGSL9* genes were upregulated in NIPP and downregulated in HTS. Moreover, the *OsGSL2*, *OsGSL4*, and *OsGSL5* genes were only highly induced in HTS in response to HS (Figure 4). Interestingly, we found that the response of *OsBGs* to HS may be more severe than that of *OsGSLs* to heat stress, and most of the *OsBGs* were upregulated in NIPP but downregulated in HTS. This phenomenon might have led to the different callose accumulation in the cells under HS.

### 2.6. Effect of Heat Stress on the Callose Accumulation and Plasmodesmata Ultrastructure of Rice Leaf

**Figure 5:**
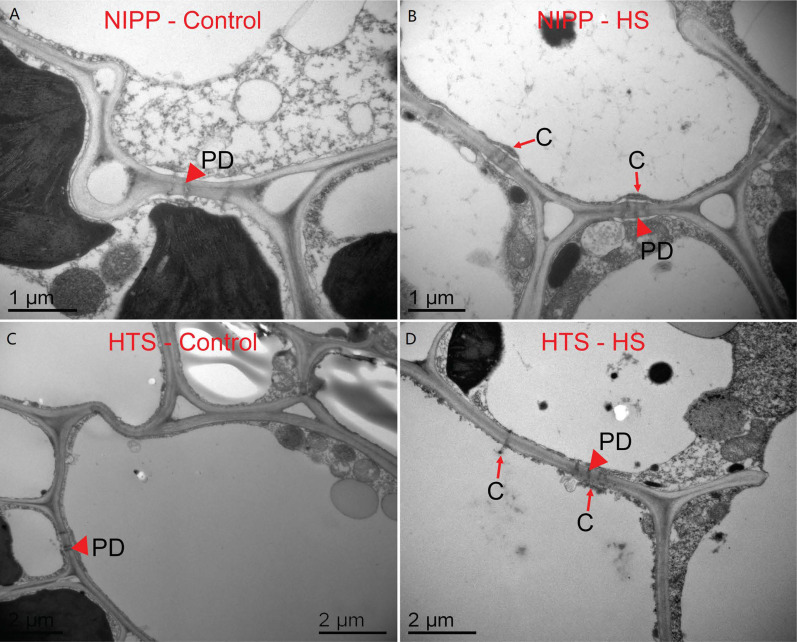
Effects of heat stress on the plasmodesmata ultrastructure in the leaf of rice at 22 DAA. (**A**,**B**) Leaves of NIPP under control (**A**) and heat stress (**B**). (**C**,**D**) Leaves of HTS under control (**C**) and heat stress (**D**).

The plasmodesmata ultrastructure of rice leaf was investigated after 10 days of HS in rice seedlings (Figure 5). The results showed that the plasmodesmata ultrastructure was slightly affected by HS. However, under HS, the callose was deposited on the surface of the plasmodesmata and could block the loading of assimilates into the leaf phloem compared with their respective controls. Moreover, more callose in the plasmodesmata was observed in HTS plants than in NIPP plants.

### 2.7. Dry Matter Distribution and Yield Analysis

**Figure 6:**
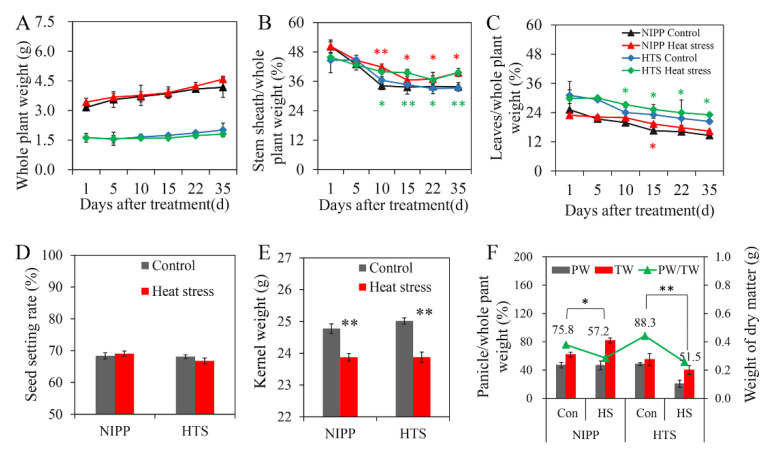
Dry matter distribution and yield measure analysis under heat stress. (**A**–**C**) Dry matter distribution of whole plants (**A**), stem sheath (**B**), and leaves (**C**). (**D**) Seed setting rate of NIPP and HTS under heat stress. (**E**) Kernel weight of NIPP and HTS under heat stress. (**F**) Panicle weight ratio of NIPP and HTS under heat stress. Vertical bars denote standard deviations (n = 3). A *t*-test was conducted for data to compare the difference between control and heat stress within one cultivar on the same day. Red asterisks: NIPP control and heat stress compared, green asterisks: HTS control and heat stress compared. * denotes *p* ≤ 0.05, ** denotes *p* ≤ 0.01.

Considering that the distribution of dry matter accumulation could help explore the rule of sugar transport in plants, the dry matter weights of the whole plant, leaves, stem, and panicle were surveyed in this study (Figure 6). According to the result, HS had little effect on the dry matter weight of the whole plant, but the distribution of dry matter changed substantially in NIPP and HTS. The ratios of the dry matter weights of the leaves and sheaths to that of the whole plant increased remarkably, but the ratio of the dry matter weight of the panicle to that of the whole plant decreased (Figure 6A–C). Accordingly, a larger decrease was observed in HTS than in NIPP under HS compared with the corresponding control (Figure 6A–C). However, HS at the grain-filling stage had little effect on the seed setting rate compared with the control. Additionally, HS considerably decreased the kernel weight by 3.65% and 4.54% in NIPP and HTS, respectively, compared with the corresponding control (Figure 6D–F). The distribution of dry matter accumulation could help explore the rule of sugar transport in plants.

## 3. Discussion

### 3.1. High Temperature Could Lead to Callose Synthesis in the Leaves of Rice

In this study, we found that 116 DEGs were enriched in the carbohydrate metabolic process item that mean high temperature could influence sugar metabolic or transport in rice. In these 116 genes, we identified 9 *BGs* that mainly contributed to the callose metabolism. Thus, high temperature may influence the yield through sugar transport; this was also argued by other related studies [26]. The expression level analysis of family members of *BGs* and *GSLs* indicated that high temperature could lead to substantial changes in the expression levels of *BGs* and *GSLs*, which could influence the callose content of leaf cells. The expression level analysis between high-tolerance NIPP and low-tolerance HTS indicated that *OsGSL2*, *OsGSL4*, and *OsGSL5* may be responsible for the higher callose biosynthesis in the low- and high-temperature tolerance varieties. The submicroscopic images of the leaf cells also indicated that after the high-temperature treatment, NIPP and HTS contained more callose than CK. This difference may be an adaption for the plant to enhance its resistance to the environment, but in terms of this change for the crop, it harmed the yield and quality of rice production. Therefore, balancing the synthesis of callose during the HS was essential. Meanwhile, exploring the function and expression patterns in future works is important for the HS breeding of rice and food security. 

### 3.2. Callose Induced by High Temperature in Leaf May Be a Factor of the Yield Decrease

Submicroscopic analysis of leaf cells under HS showed that more callose was synthesized, and the plasmodesmata were blocked up. The plasmodesmata are the main means of endoplasmic sugar transport. These blockages show mainly the low efficiency of the non-smooth flow. Considering the sugar transported in whole plants, less sugars were transported to the grains, more sugars remained in the leaves, and the sugar allocation ratio of different organs was strongly influenced. This unbalanced transport will change the morphogenesis of plants and decrease the yield and quality of production. All these results implied that high temperature could lead to the higher expression of *OsGSL2*, OsGSL4, and *OsGSL5* in lower-tolerance HTS. These genes help to generate more callose in the cell and block up the plasmodesmata. This phenomenon was also found in citrus leaves when infected with *Candidatus* Liberibacter asiaticus [13]. The obstructed plasmodesmata could limit the sugar transport through the endoplasmic way in leaves and finally lead to the decrease in yield and quality. The dry matter weights and yields of the CK and HS plants also indicated that HS influenced the dry matter distribution. This result was also consistent with previous studies in the physiological level, which confirmed that high temperature could increase the dry matter rate of leaves and decrease the yield of grains [29]. Hence, for the sake of surviving extreme hot weather in the future, breeding varieties with low- or high-temperature-induced callose generation or the adoption of new cultivating methods to decrease the influence of callose may be necessary.

### 3.3. Gain or Loss of BGs and GSLs May Contribute to Environmental Adaption

Three ecotypes of *Oryza* were considered in this study to explore the family members and evolution relationship of the two families. The phylogenetic and synteny analyses of these members indicated that the gene gain and loss of *BGs* and *GSLs* occurred after the divergence of the three ecotypes. *OS* gained more genes and *Os* lost more genes than the other ecotypes. This finding indicates an expansion in the *OS* ecotype, which could enhance callose metabolism and increase adaptation to a high-temperature environment [30,31,32]. *OS* exists and is cultivated in South China, Southeast Asia, and some other high-temperature areas, and *Os* exists in the temperate zone of the world [33,34,35,36]. Therefore, these gains and losses of family members might be an environmental adaption that occurred during long-time evolution after divergence from a common ancestor. These gains and losses contributed to their environmental adaptability and also lead to the diversity of their genotypes and phenotypes. Thus, all these ecotypes were important for rice abiotic breeding and resistance studies.

## 4. Materials and Methods

### 4.1. Plant Materials and Growth Conditions

The study was conducted at the experimental farm of Shandong Agricultural University, Taian, Shandong Province. The two rice genotypes selected in this study were NIPP and HTS, which have different heat tolerances. The rice seeds were sown in pots filled with paddy soil. Seedlings with 4–5 leaves were divided into two groups and moved into two separate plant growth chambers with controlled temperature and relative humidity. One group of rice plants was subjected to high-temperature treatment (40 °C from 09:00 to 16:00, and 30 °C from 16: 01 to 8: 59 of the next day for 6 days), and the other one set was set as the control (30 °C from 09:00 to 16:00, and 24 °C from 16.01 to 8:59 of the next day for 6 days). Both groups were maintained under 70–80% relative humidity and natural sunlight conditions.

### 4.2. Real-Time Quantitative PCR

For quantitative real-time PCR analysis, samples were prepared from leaves harvested 6 days after heat stress. The samples were frozen in liquid nitrogen and stored at −80 °C. Total RNA was extracted from the samples using TRIpure reagent (Aidlab Biotechnologies, Beijing, China). RNA was converted into first-strand cDNA using the ReverTra Ace qPCR RT Master Mix (TOYOBO, Shanghai, China) and oligo (dT) as the primer. The resultant cDNA was used as the template for quantitative PCR amplification in a Thermal Cycler Dice Real-Time System II (TaKaRa Biotechnology, Dalian, China) with SYBR Green I (TOYOBO) as the fluorescent reporter. Primers were designed to generate 150–250 base pair (bp) fragments using the PRIMER3 software [37] (Table S3). PCR analysis and detection were performed as described previously [38]. The 2^−ΔΔCT^ method was employed to analyze the relative gene expression levels using the mean values from three replicates.

### 4.3. Transmission Electron Microscopy and Scanning Electron Microscopy

Leaf samples were collected 6 days after HS. For transmission electron microscopy analysis, the samples were first fixed with 3.5% glutaraldehyde in phosphate buffer (0.1 M, pH 7.0) for about 6 h and then washed three times in phosphate buffer for 15 min. Lastly, these specimens were post-fixed with 1% OsO_4_ in phosphate buffer for about 1–2 h and washed three times in the phosphate buffer for 15 minutes. The tissues were dehydrated in a graded ethanol series (30%, 50%, 70%, 80%, 90%, 95%, and 100%) for 20 min each step and slowly and progressively infiltrated with Spurr’s resin, mixed with acetone, and infiltrated up to 100% Spurr’s resin overnight. The specimens were placed in Eppendorf tubes containing Spurr’s resin and heated at 70 °C for more than 9 h. The specimens were sectioned in a LEICA EMUC 7 ultramicrotome, and the sections were stained with uranyl acetate and alkaline lead citrate for 5–10 min each and then observed on a Hitachi Model H-7650 transmission electron microscope.

### 4.4. RNA-Seq Analysis

The RNA extraction, library building, and sequencing were processed by the Yijiyin Technology Company (Hangzhou, China). The raw data were generated by Illumina Hiseq400 platform with the system PE150 System. The obtained raw data were first cleaned and trimmed by Trimmomatic (v0.36) [39]. Then, the raw data were mapped on to the reference genome of *O. sativa* subsp. *japonica* (v7.0) [40] by Tophat2 [41]. The expression level and DEGs were processed by Cufflinks and Cuffdiff [42]. The DEGs were finally selected according to the cutoffs: false discovery rate <0.05 and |log2(change fold)| > 1.

### 4.5. Identification of the Family Members

Two methods were used to identify the family members in three ecotypes [40,43,44]. The first method was using the HMMSEARCH [45]. The *GSL* model was downloaded from Pfam (no. PF02364.13) [46], and the *BG* model was built on the basis of the aligned sequences download from NCBI [47]. The second method was based on BLASTP [48]. All protein sequences belonging to three ecotypes were scanned against the downloaded protein sequences, and the hits with *e* < ×10^−5^ were retained. Finally, all the candidate genes were combined and verified by NCBI-CDD search (https://www.ncbi.nlm.nih.gov/Structure/cdd/wrpsb.cgi, accessed on 8 October 2020) [47]. Only the genes that contained the expected domain were considered the family members. Sequence alignment and phylogenetic analysis were processed by MEGA X with the model of neighbor join and bootstrap of 1000 [49]. Whole genome duplication analysis was processed by MCScanX [50], and the visualizations were realized by CIRCOS [51].

### 4.6. Statistical Analyses

The SPSS 11.5 software was used to determine the significance of the differences be-tween or among the data. Three replicate experiments were carried out in order to analyze the mean values and standard errors in the figures represent data. A *t*-test was conducted for data provided in Figure 4 and Figure 6 in order to compare the difference between control and heat stress. One way analysis of variance (ANOVA) was conducted to compare the difference with a least significant difference test (LSD) at *p* ≤ 0.05 for the data. * denotes *p* ≤ 0.05, ** denotes *p* ≤ 0.01.

## 5. Conclusions

In this study, we found that heat stress changed the expression patterns of *BGs* and *GSLs* in rice leaves. This expression piled up the callose in cells, which blocked the plasmodesmata and finally decreased the sugar transport from the symplast pathway. We preliminarily explored the mechanism of the decrease in yield under HS and provided many new insights into the environmental adaption and sugar transport during HS. The study will benefit heat tolerance research, as well as breeding and cultivation measures, in the future.

## Data Availability

The raw data of RNAseq has been deposited to National Genomics Data Center (NGDC, https://ngdc.cncb.ac.cn/) with the project number of PRJCA014715.

## Supplementary Materials

The following supporting information can be downloaded at: https://www.mdpi.com/article/10.3390/ijms24043175/s1.
